# Editorial: Microbiome dynamics as biomarkers of welfare status in aquatic species

**DOI:** 10.3389/fphys.2023.1276351

**Published:** 2023-08-22

**Authors:** F. Fonseca, J. Fuentes

**Affiliations:** ^1^ Centro de Investigação Marinha e Ambiental (CIMA), University of Algarve, Faro, Portugal; ^2^ ARNET-Aquatic Research Network-Associate Laboratory, University of Algarve, Faro, Portugal; ^3^ Centre of Marine Sciences (CCMar), Universidade do Algarve, Faro, Portugal

**Keywords:** microbiome dynamics, biomarkers, welfare status, aquatic species, aquaculture probiotics

The importance of the microbiome in aquaculture species is widely acknowledged. The fish gut microbiota is essential for host fitness, modulated by diet and environment, and affects animal physiology. The gut microbiome optimizes nutrient uptake and immunomodulation and enhances host resilience against infectious diseases. Understanding microbiome dynamics in aquaculture species is vital in promoting animal health and industry sustainability.

In this Research Topic of Frontiers four original papers and one review paper were accepted. This editorial provides an integrated summary of those papers’ main discoveries and clues.

Two of the papers address mainly the problem of nitrogenous production/accumulation in rearing systems, especially with carnivorous fish species, like common carp and grouper, and strategies to mitigate toxicity and reduce the nitrogen load in aquaculture conditions. The amount of ammonia that fish produce and excrete responds to various factors, including their food intake and the protein content of their diet. Researchers have found that fish can host nitrogen-cycle bacteria in their gill cells, which can aid in the production of dinitrogen gas (N_2_) and reduce the amount of ammonia excretion caused by feeding. Additionally, studies show that carp excrete less ammonia when fed on demand compared to being fed the same amount twice daily by hand. In the study by Mes et al. comparing the growth and nitrogen excretion of demand-fed carps with batch-fed carps, the authors found that the feeding regime had a significant impact on nitrogen balance and that carp can excrete N_2_ at a rate in the same order of magnitude as the amounts of nitrogen that were found unaccounted for in the calculated balance. The authors suggest that N_2_ production by symbiotic branchial nitrogen cycle bacteria is likely a universal feature that needs to be considered to fully understand nitrogen balance in fish and the functional role of their microbiome.

The accumulation of toxic nitrogen forms in rearing environments is a significant concern in intensive aquaculture systems. Asian fish farmers commonly use fishponds to rear grouper, relying on stable water quality achieved by using environmental heterotrophic bacteria to break down excess waste. However, overfeeding or keeping fish at high densities can increase organic matter to dangerous levels by accumulating ammonia and nitrites, which makes crucial the development of pond culture technology that meets farmers needs and ensures animal health. In the study by Huang et al. *Bacillus* sp. and *Lactobacillus* sp. were added to rearing water to evaluate their potential probiotic beneficial effects on microbial communities and overall water quality. The authors observed a significant decrease in ammonia and nitrite concentration in prebiotic-treated groups throughout the 22-day feeding period. At the higher concentration treatment, probiotics altered the composition of the microbial community by significantly increasing the abundance of Planctomycetes while significantly decreasing the abundance of Fusobacteria. Bacteria in phyla Planctomycetes, typically recognized as the ANAMMOX type, benefited from the organic acid produced by the probiotic bacteria, while Fusobacteria such as *Cetobacterium* spp. (related to protein digestion and vitamin B12 production) failed to compete for resources. Including probiotics led to better results in fish growth parameters in the treated groups. Although this study strongly suggests the bioremediation capacity of probiotics, it also makes obvious the necessity to conduct further studies to fully comprehend and evaluate the extent of the dysbiosis effect on the natural microbial community in the water and fish gut.

In the paper by Liao et al. the authors studied the effects of the “Fishery-photovoltaic Integration” (FPVI) aquaculture system on the growth rate and ecological environment of shrimp, using *Penaeus monodon* as a model species. They characterized the bacterial community structures in water, effluent, and intestinal environments using 16S gene sequencing. The results showed that the microbiota composition under the FPVI system promotes the healthy growth of shrimp by regulating intestinal function and preserving intestinal homeostasis. Additionally, the microbiota regulates nitrogen transformation in the water, ensuring the stability of the water environment throughout the 105-day experiment period with a reduction in antibiotic resistance genes observed within the FPVI pond. The findings suggest that the FPVI aquaculture model holds significant potential for future development and testing with other aquaculture species and rearing systems.

A higher diversity of gut microbes leads to better metabolic health in humans and other mammals. Exercise, even in moderation or short-term, can diversify intestinal microorganisms and restore the balance of beneficial and harmful microbes. The use of motor stimulation in fish aquaculture as part of environmental enrichment strategies is a current concern. Wei et al. studied the effect of exercise on a novel hybrid of *Megalobrama amblycephala* (female) and *Ancherythroculter nicrocauda* (male), which showed quick growth, disease resistance, and anti-stress capability but needed improvement in fillet quality. By allowing the fish a short-term (12–16 days) swimming exercise at a constant moderate speed in circular flumes, the authors observed increased muscle fibre density, decreased muscle fibre diameter, and improved muscle texture. The exercise significantly increased gut bacterial community richness and diversity and altered the composition of gut microflora, as confirmed by 16S gene sequencing analysis. At the phylum level, the relative richness of Proteobacteria, Firmicutes, and Bacteroidetes was increased in the exercised groups, while that of Fusobacteria decreased. Functionally, these alterations can boost glucose, nitrogen cycling, metabolism, and the cycling of protein-rich compounds. Including the reported alterations at the genus level, the results suggest that short-term swimming exercise also increases intrinsic antimicrobial resistance and promotes the denitrification capacity of this hybrid species.

Finally, the review paper by Kumar et al. opened other perspectives to studying fish microbiota, using Zebrafish (*Danio rerio*) as a model species in the context of human pathologies. In a comprehensive survey of the literature, the authors highlight aspects that justify the use of Zebrafish as a valuable tool to identify microbial enterotypes in fish and humans and investigate the link between the gut microbiome and physiological homeostasis of cardiovascular, neural, and immune systems. Zebrafish can be reared germ-free under gnotobiotic husbandry practices, with the apparent advantages of directly investigating specific host-microbiota interactions. The review demonstrates the significant overlap between Zebrafish and mammalian gut microbiota composition and the physiological responses of gut bacteria in both models. The paper supports the idea that sustained improvements in methodology to unravel host-microbe interactions using Zebrafish as a tool will benefit both mammalian and aquatic microbiota research.

